# MinION Nanopore Sequencing Enables Correlation between Resistome Phenotype and Genotype of Coliform Bacteria in Municipal Sewage

**DOI:** 10.3389/fmicb.2017.02105

**Published:** 2017-10-31

**Authors:** Yu Xia, An-Dong Li, Yu Deng, Xiao-Tao Jiang, Li-Guan Li, Tong Zhang

**Affiliations:** Environmental Biotechnology Laboratory, The University of Hong Kong, Pokfulam, Hong Kong

**Keywords:** MinION, ARGS, fecal coliforms, sewage, nanopore, resistome

## Abstract

Wastewater treatment plants (WWTPs) functioned as the intersection between the human society and nature environment, are receiving increasingly more attention on risk assessment of the acquisition of environmental antibiotic resistance genes (ARGs) by pathogenetic populations during treatment. However, because of the general lack of robust resistome profiling methods, genotype, and resistance phenotype is still poorly correlated in human pathogens of sewage samples. Here we applied MinION sequencing to quantify the resistance genes of multiple antibiotic resistant (MAR) coliform bacteria, a common indicator for human enteric pathogens in sewage samples. Our pipeline could deliver the results within 30 h from sample collection and the resistome quantification was consistent to that based on the Illumina platform. Additionally, the long nanopore reads not only enabled a simultaneous identification of the carrier populations of ARGs detected, but also facilitated the genome reconstruction of a representative MAR strain, from which we identified an instance of chromosomal integration of environmental resistance gene obtained by plasmid exchange with a porcine pathogen. This study demonstrated the utilization of MinION sequencing in quick monitoring and simultaneous phylogenetic tracking of environmental ARGs to address potential health risk associated with them.

## Introduction

With the spread of antibiotic resistant pathogens and the related increase of human morbidity and mortality, antibiotic resistance genes (ARGs) have been considered as emerging environmental pollutants by World Health Organization (2014). Recent metagenomic techniques based studies have shown that putative ARGs were present in nearly all ecosystems (Sommer et al., 2009; Allen et al., 2010; Chen et al., 2013, p.20; Hu et al., 2013; Hatosy and Martiny, 2015). Given the ubiquitous nature of environmental ARGs, the potential resistance acquisition by clinical pathogens in environmental settings is a subject of ongoing concern. Direct contact between pathogenic bacteria and environmental ARG carriers, plus a constant selective pressure imposed by traces of antibiotics in the sewage, makes domestic wastewater system an ideal hub for horizontal transfer of ARGs between microorganisms (Renew and Huang, 2004; Brown et al., 2006; Watkinson et al., 2009). Though some researchers found the core resistome of wastewater treatment system was different from that of human pathogens (Munck et al., 2015), wastewater treatment plants (WWTPs) are receiving increasingly more social and scientific attention on risk assessment of the acquisition of environmental ARGs by clinical pathogens during the treatment processes (Finley et al., 2013; Yang et al., 2014).

Among the microbial communities of WWTPs, coliform bacteria are the most commonly used indicator for sanitary quality of water before and after treatment and fecal coliforms have frequently been surveyed as indicator of potential presence of human enteric pathogens (Meng et al., 2015). In water samples, fecal coliforms (from animal and/or human sources) could be measured as multiple antibiotic resistant (MAR) coliforms (Hagedorn et al., 1999; Simpson et al., 2002; Dhiman et al., 2016). Therefore, investigation on the resistome (composition of ARGs) of MAR coliforms would add valuable genetic information to facilitate the risk assessment of environmental ARGs in wastewater treatment processes.

Compared to qPCR methods, sequencing-based ARGs detection benefits a more authentic quantification by avoiding the intrinsic limitation of ARG primers. As a result, ARG detection based on high-throughput shotgun sequencing was increasingly applied to study the distribution of environmental ARGs in different ecosystems, including wastewater treatment systems (Freilich et al., 2010; Li et al., 2015). However, majority of the sequencer (e.g., Illumina platforms, 454 platforms, PacBio platforms) could hardly achieve the real-time resistome profiling which is required to guide the resistance control measures. Furthermore, the short read length and the fragmented assembly make it challenging to identify the carrier populations of ARGs based on these massive sequencing platforms.

DNA sequencing using nanopore technology by Oxford Nanopore Technologies Ltd. is an alternative method for rapid generation of long-read sequences. Genes acquired by horizontal gene transfer, such as resistance genes, usually have an inherent repetitive nature due to being flanked by or interspersed with insertion sequences, which will leave numerous gaps in the short-read assemblies. The long reads produced by MinION sequencing could resolve the connection among repetitive regions and facilitate whole genome assembly of the host of the resistance genes (Ashton et al., 2014).

Additionally, MinION sequencing may provide a time-saving and portable framework, which could reduce the time required from sample collection to results delivery, making it possible to adopt a real-time rapid protocol in resistome monitoring practice (Quick et al., 2016). Although the error rate of MinION sequencing leaves an open question whether it is feasible for such a purpose. Many specially designed bioinformatic algorithms could solve the fundamental computational challenges for error correction and *de novo* assembly of nanopore long reads (Loman and Quinlan, 2014). van der Helm et al. utilized MinION sequencing to quantify the resistome of functional selected metagenomic libraries (van der Helm et al., 2017). However, the application of these tools in resistome detection of environmental samples still remained poorly understood.

In this study, MinION sequencing was applied to investigate the resistance genes carried by the MAR coliform bacteria cultured from the influent of a local wastewater treatment plant (WWTP) (Shatin, Hong Kong, SAR, China). The pipeline of this study was summarized in Figure S1. We set out to develop methods to explore the taxonomic information and predict the resistance profile, especially ARGs on mobile genetic elements, carried by these fecal coliforms. The resistance profile predicted using MinION platform was also compared to that based on short-read Illumina technology. Additionally, we reported a hybrid assembly of combined MinION and Illumina data to identify the structure and insertion site of a chromosomal antibiotic resistance island in a pathogenic MAR coliform strain, revealing resistant plasmids shared between coliforms in WWTP and human and porcine pathogens.

## Materials and methods

### Sample collection

Sewage sample was collected from the influent channel after the grit chamber at Shatin WWTP located at Hong Kong, SAR, China. The sewage sample was transported to the laboratory within 1 h after sampling and handled immediately for coliform bacteria culturing.

### Isolation of multi-drug resistant coliform bacteria

To provide multi-drug enriched environment, combined antibiotics of ampicillin, kanamycin, and chloramphenicol (Composition shown in Table S1) were added to the MacConkey and Lysogeny broth (LB) agar. The sewage sample was diluted in ultrapure water by 100 times for the cultivation of coliform bacteria. 100 μl of the diluted sewage sample was spread on to the multi-drug modified MacConkey agar plate. After incubating overnight (10 h) at 37°C, the multidrug resistant coliform isolates were randomly picked and inoculated into 200 μl LB media on 96-well plates and cultured at 37°C with shaking at 200 rpm for another 10 h. One of the coliform clones, named as COL1, was randomly picked out for whole genome sequencing on both MinION and Illumina Hiseq2500 platforms. All the randomly picked colonies, including COL1, were harvested when their OD600 reached 1.2 in LB media with the target antibiotics at 37°C. The COL1 colony was re-cultured against ampicillin, kanamycin, tetracycline, erythromycin and chloramphenicol to confirm its resistance phenotype.

### DNA extraction and illumina sequencing

Ninety-six randomly picked coliform isolates were firstly mixed with equal ratio (30 μl culture per isolates) before DNA extraction. Total DNA of the coliform isolates mixture and the COL1 strain was, respectively extracted using FastDNA® Spin Kit for Soil (MP Biomedicals, USA) following the manufacturer's instructions. Extracted DNA was quantified using Qubit 2.0 Fluorometer (Invitrogen Life Technologies) (Table S2). Next, the extracted DNA was sent out for high-throughput metagenomic sequencing on the Illumina Hiseq2500 platform using the PE125 strategy (Paired-end sequencing with 125 bp read length) at the Groken Bioscience Limited (Hong Kong, SAR). (Besides the ones used for DNA extraction, the isolates were then sent for autoclave. Additionally, all the experiments were operated in biological safety cabinet).

### MinION library preparation and sequencing

The sequencing library was individually prepared for coliform isolates and the COL1 strain using Oxford Nanopore MinION Genomic DNA Sequencing Kit (SQK-MAP006) (See supporting information for detailed library preparation protocol). Library concentration after each preparation step can be found in Table S2.

After inserting flow cell (R7.3 Chemistry) into the MinION device, a flow cell quality control protocol was run to assess pore activity: 309 and 180 single pores were respectively detected for the flow cells used for MinION sequencing of the coliform isolates and the COL1 strain. Next, flow cell was equilibrated before loading the sequencing library gently via sample port. Two independent 6 h MinION runs were conducted, respectively for coliform isolates and COL1 strain. The MinION libraries and corresponding Illumina datasets has been submitted to ENA at study accession PRJEB20722.

### Plasmid resistance

Because metagenomic sequencing indicated the presence of plasmids in clone COL1, plasmids in the COL1 clone were extracted using QIAGEN Plasmid Mini Kit (QIAGEN Science Inc., USA) following the manufacturer's protocol. To determine the antibiotic resistance conferred by the extracted plasmid, the extracted plasmids were transformed into the antibiotic sensitive competent *E. coli* DH5α cell. Next, the transformed DH5α was inoculated onto antibiotic-containing LB plates to test the antibiotic resistance of the target plasmids. Composition and concentration of antibiotics used in the antibiotic-containing LB plates were the same with that applied in the isolation step (Table S1).

### Close the circular structure of putative plasmids

PCR was carried out with the attempt to close the circular structure of the incomplete putative plasmids of NODE5 (160 kb) and NODE7 (40 kb) identified in the assembly. Primers were designed based on the assembly results (see Table S3 for primer sequences designed). See Supporting Information for the PCR systems and condition used for the amplification. The PCR products were sent out for Sanger sequencing at BGI-Shenzhen (BGI, China). However, as multiple sequences (Figure S2) remained in the amplified band of NODE5, Sanger sequencing of NODE5's amplicon failed after multiple tries. These non-specific amplifications in NODE5 were probably caused by repetitive insertion on the plasmid.

### PCR verification of inconsistent ARG subtypes between illumina and nanopore datasets

Five ARG subtypes identified only in Illumina or nanopore datasets were selected for validation by PCR. Five pairs of primers were designed by Primer3 (Untergasser et al., 2012, p. 3) (Table S4) to verify the ARG subtypes using 50 μl PCR reaction systems of: 25 μl 2 × PCR premix (Takara, China), 1.5 μl forward primer, 1.5 μl reverse primer, 1 μl extracted coliform metagenome DNA, and 21 μl of nuclease free water. PCR conditions used for the amplification includes: initial denaturation at 95°C for 5 min, followed by 35 cycles of denaturing at 95°C for 30 s, annealing at 55°C for 30 s, and extension at 72°C for 1 min; and finally, extra extension at 72°C for 10 min.

### Data analyses

For both nanopore datasets of coliform isolates and COL1 strain, Poretools (Loman and Quinlan, 2014) were used to extract 2D reads from fast5 data obtained by MinION sequencing. Only fast5 files passing the quality filter by the 2D Basecalling pipeline on Metrichor were used for 2D reads extraction.

### Error estimation of nanopore 2D reads

To estimate the error rate of MinION sequencing, the extracted 2D reads were mapped to the corresponding *do novo* assembly of Illumina data (by CLCbio Genomic workbench with default parameters) using LAST aligner with parameters of “lastal -s 2 -T 0 -Q 0 -a 1.” Miscalled bases were determined by parsing the alignment output taking Illumina assembly as golden standard. To identify k-mers that have a higher tendency of being missed by MinION sequencing, all deletions of length k (where k equal to 1 to 6 base pair) in the nanopore reads were extracted from the mapping file to Illumina assembly using SAMtools (Li et al., 2009). Z-score was calculated as z = (x-μ)/σ, where × is the proportion of a k-mer across all deleted k-mers of that length, μ is the mean and σ is the standard deviation for the proportion of deletions across all k-mers of that length. A similar process was used to analyze the inserted k-mers.

### Hybrid assembly of nanopore and illumina sequences of the COL1 strain

Hybrid assembly of Nanopore and Illumina sequences were performed using SPAdes (version 3.6.2) (Bankevich et al., 2012) with –careful and –nanopore flag. Next, extension was conducted to scaffolding the derived assembly following previous protocol (Ashton et al., 2014). For comparison, Nanopore sequences were individually assembled by Canu (Koren et al., 2017) with error rate of 0.035, while the post-QC Illumina reads were assembled by CLC bio Genomics Workbench (version 6.0.4, CLC Bio, Denmark) with default parameters. To avoid the interference of fragmented assembly, contigs shorter than 1 kb or with coverage <20 were filtered before annotation.

### Plasmid identification in hybrid assembly of COL1 strain

Putative plasmids were picked out for manual check if contigs satisfy any of the criteria: (1) circular sequence overlapped for at least 50 bp at the two ends of a contig; (2) over 50% length of the contig showed >90% similarity to known plasmids sequences in NCBI *nt* database by BLASTN (Camacho et al., 2009) search; (3) circular structure could be verified by PCR amplification.

### Annotation and genome comparison of COL1 genome

Genome sequence of COL1 strain was submitted to NCBI Prokaryotic Genomes Annotation Pipeline for gene calling and annotation (Accession PRJNA318310). To further confirm annotation of ARGs, the predicted amino acid sequences were further BLASTP v2.2.28 + (Camacho et al., 2009) against SARG database (combining ARG sequences of ARDB and the CARD v1.1 with manual curation on ARG types and subtypes based on resistance mechanisms) (Yang et al., 2013) with similarity cutoff 60% and alignment length cutoff of 50% (Yang et al., 2013; Ma et al., 2016). Ribosome RNA genes were double confirmed by online RNAmmer 1.2 server (Lagesen et al., 2007). The derived 16S rRNA genes were searched against Silva SSU Ref non-redundant (NR99) 119 database (Quast et al., 2013) for phylogenetic assignment. To identify the genomic features divergent from closely related strains, BRIG v0.95 (Alikhan et al., 2011) was used to map reference genome sequences to the COL1 genome with default BLASTN v2.2.28 + parameters. Reference genomes were selected if their genome or plasmid sequences were among the closest matches to 16S rRNA genes or plasmid sequences of COL1 strain. IslandViewer 3 (Dhillon et al., 2015) was used to identify the gene islands on the COL1 genome based on the WGS annotation. PHAST (Zhou et al., 2011) was used to detect phage insertion sequences on the genome of COL1.

### Taxonomy classification of nanopore reads for the coliform isolates

Identification of bacterial species presented in the coliform culture was carried out based on alignment of Nanopore 2D reads to MetaPhlAn 2 database of taxon-defining marker genes (Segata et al., 2012). Alignment of Nanopore 2D reads was performed with LAST tool (version 744) (Frith et al., 2010), invoking lastal with custom settings as -a 1 -b 1 -q 2 (Quick et al., 2015). LAST package was selected here because it can align sequences with tolerance to many mismatches and gaps. Only the best alignment (with the highest bit score) showing similarity higher than 60% over more than 60% of the marker gene length was kept for taxonomy classification. Relative abundance of a population was calculated as the number of 2D reads assigned to this taxon in percentage of the total number of annotated 2D reads could be assigned at a given taxonomic level.

### ARG identification on nanopore reads of both coliform isolates and COL1 strain

2D reads derived from MinION sequencing was aligned to the nucleotide sequences of SARG database (Yang et al., 2013). Alignment of Nanopore 2D reads was performed with LAST tool (version 744) (Frith et al., 2010) with custom settings as -a 1 (Ashton et al., 2014). Next, tabular result of LAST search was filtered based on alignment length and similarity. Only alignment showing alignment length >50% of ARG length with similarity >60% was used for ARG counting. If ARG-containing regions on Nanopore reads overlapped for more than 80% of the alignment length, only the best ARG hit (with the highest bit score) was kept for this overlapped region. The concept of ppm introduced by Yang et al. (2013) (base pair of ARG in million base pairs of sequence) was used for the quantification of ARG level in the coliform sample.

### ARG quantification based on illumina sequencing

Antibiotic resistance genes (ARGs) in coliform culture were also quantified based on Illumina datasets following previous protocol. Briefly, post-QC short reads were firstly searched against the SARG database with BLASTX with *E*-value cutoff of 1E-5 (Minot et al., 2011). Next, the alignment was filtered allowing ≥90% amino acid identity and ≥25 amino acids alignment length (Yang et al., 2013). For the easy of comparison, ARGs were quantified in term of ppm as previously stated.

## Results

### Error estimation of nanopore reads

Six hour run of MinION sequencing resulted in a total of 12,047 effective 2D sequences with median length of 5,796 bp, a maximum length of 18,582, median accuracy of 84.6% (estimated based on alignment to Illumina assembly) and a total of 58.0 Mb of data (Table S5) for both the resistance profiling and the assembly of the coliform isolate chromosome isolate. Based on the mapping error-profile of MinION reads to Illumina assembly, we observed an indel size of 3.8 Mb in the 56.7 Mb mapped 2D reads. 60.1% of these indels were deletions (2.3 Mb), while insertion took 1.5 Mb. This indel frequency (6%) was 50% lower than that reported by R7.0 chemistry (indel frequency of 17.2 and 22.2%, respectively by Ashton et al., 2014 and Judge et al., 2015). These results suggested indel error was reduced by the R7.3 flow cell chemistry. However, it is noticed that kmers with extreme GC content of 0 and 100% still showed apparently higher-than-expected tendency for deletion and insertion (Figure S3). Additionally, we observed short palindrome (sequence with nucleic acids reading forwards matches the complementary sequences reading backwards) 6-mer of TCTAGA showed particularly high deletion frequency (z-score > 20) suggesting the current library protocol may have difficulty to fully denature the short hairpin structure caused by the palindrome sequences (Figure S3).

### Rapid resistance gene detection in the MAR coliforms

To rapidly determine the resistome carried by fecal coliforms in sewage sample, MAR coliform bacteria was cultured from sewage sample of a local WWTP following standard protocol. Cultured with high concentration of antibiotics (Table S1) is used for the enrichment of the ARGs in the target sequenced dataset, which made the genotype analyses more straightforward. Next, 6 h MinION run was conducted with DNA library freshly prepared using DNA of coliform isolates, resulting in 3,535 2D reads with median length of 2,503 bp (Table S5). Based on the 2D reads derived, a bioinformatics pipeline was developed to predict the resistome in term of ARG abundance and populations carrying these ARGs. The whole process, including sample collection, MAR coliforms culturing, library preparation, MinION sequencing, subsequent data processing and finally resistome prediction, were finished within 30 h.

Antibiotic resistant genes were identified from these 2D reads if a valid alignment to a reference ARG sequence in the SARG database could be found. The valid ARG alignments of Nanopore 2D reads showed a median alignment length of 1,078 bp with average similarity of 78% (Figure S4). Therefore, despite the error rate (9% based on Phred Score and 15.4% based on mapping to Illumina assembly), credible ARG counting could still be achieved as the ARG-carrying nanopore sequences showed length of exact matched base pairs (on average >800 bp) almost equal to the whole length of reference ARG sequences in SARG database (1,131 bp on average). 119 ARGs belonging to 15 different antibiotic types were identified on 97 Nanopore 2D reads (which was equal to 2.7% of all the 2D reads). The inherent advantage of long sequence length generated by the MinION platform provides a unique opportunity for simultaneous host tracking for environmental ARGs which is indispensable to truly assess the risk associated with them (Bengtsson-Palme and Larsson, 2015; Martínez et al., 2015). Consequently, the carrier populations of these ARGs were determined by searching the long ARG-carrying Nanopore reads against the metagenomic marker gene database of MetaPhlan 2, containing unique marker gene catalogs specific to a phylogenetic lineage down to species level. Almost half of these ARG-carrying sequences (47 out of 97 2D reads) could be assigned to a known genus and 31 reads of them could be further assigned to a specific species (Table S6).

Antibiotic resistance genes (ARGs) assigned to the multidrug category (Table 1) were the most prevalent resistance gene type detected. Such prevalence of Multidrug ARGs should be in part originated from the higher presentation of this category within the SARG database (taking 22.6% of all the ARG reference sequences, Figure S5). The outer membrane protein of omp36 and ompF, allowing passive diffusion of various kinds of antibiotics across the outer membrane (Thiolas et al., 2004; Duval et al., 2009), were the most frequently identified resistance mechanism in the coliform community; however, we failed to classify the carrier populations for nine out of the 11 nanopore reads encoding this mechanism. Additionally, the DNA-binding protein Hns (regulator for multidrug exporter genes; Nishino and Yamaguchi, 2004) and histidine kinase (beta-lactam receptor inducing resistance to beta-lactam; Li et al., 2016) were the most frequently detected ARGs in the “Others” ARG category. Similarly, none of the nine carrying nanopore sequences could be phylogenetically assigned by marker gene search. In the classifiable proportion of nanopore dataset, it was interesting to notice that high concentration of *tet* (Table 1) was identified on phage of *Enterobacteria* phage HK002 suggesting potential involvement of phage transduction in the dispersion of tetracycline resistant genes in the fecal coliform community. Additionally, clinically significant human pathogens of *Klebsiella pneumonia* (Podschun and Ullmann, 1998)*, Enterobacter cloacae* (Harbarth et al., 1999), and *Serratia marcescens* (Knothe et al., 1983) were prevalent in the MAR coliform community (Table 1, Table S7), mirroring their multi-drug resistance enabled by a series of ARGs, especially the multi-drug resistant transmembrane transporter genes (Table 1).

**Table 1:** Abundance of antibiotic resistance genes and their carrier species identified in the coliform culture based on Nanopore 2D reads.

**Carrier species**	Ami^*^	β-lac^*^	Chl^*^	Mac^*^	Tet^*^	**Bac**	**Ble**	**Fosf**	**Fosm**	**Kas**	**Poly**	**Quin**	**Rif**	**Sulf**	**Trim**	**Mult**	**Others**	**Abun^a^ (*%*)**
*Klebsiella pneumoniae*	-	166(1)	-	-	33(1)	-	-	-	-	-	-	-	-	-	-	434(5)	-	26.8
*Enterobacter cloacae*	-	-	-	-	-	-	-	-	-	-	-	-	-	-	-	108(1)	52(1)	6.7
*Escherichia fergusonii*	-	-	-	-	-	-	-	-	-	-	-	-	-	-	-	744(4)	33(1)	3.3
*Serratia marcescens*	147(2)	-	57(1)	-	127(1)	-	31(1)	-	-	-	-	-	-	77(1)	-	-	-	2.9
*Enterobacteria* phage HK022	-	-	-	-	169(1)	-	-	-	-	-	-	-	-	-	-	-	-	2.0
*Enterobacter* sp MGH14	222(3)	-	-	-	-	-	-	-	-	-	-	62(1)	-	153(2)	42(1)	-	-	1.0
*Enterobacter* sp MGH24	-	-	-	-	-	-	-	-	-	-	-	-	-	-	-	61(1)	-	1.0
*Enterobacter* sp MGH26	-	207(3)	-	-	-	-	-	-	-	-	-	-	-	-	-	-	-	0.6
*Yersinia pseudotuberculosis*	-	-	107(1)	-	-	-	-	-	-	-	-	-	-	-	-	-	-	0.6
*Citrobacter koseri*	-	-	-	-	-	-	-	-	251(2)	-	-	-	-	-	-	-	-	0.4
*Pantoea agglomerans*	-	-	-	-	-	-	-	-	-	-	-	-	-	-	-	68(1)	-	0.4
*Sinorhizobium meliloti*	158(2)	-	-	-	-	-	-	-	-	-	-	-	-	-	-	-	-	0.4
*Yersinia ruckeri*	-	-	-	-	-	-	-	-	-	-	-	-	-	-	-	-	53(1)	0.4
*Acidiphilium multivorum*	-	-	-	-	-	-	-	-	-	-	-	-	-	75(1)	-	-	-	0.2
*Dehalobacter* sp CF	-	76(1)	-	-	-	-	-	-	-	-	-	-	-	-	-	-	-	0.2
Unclassified	67(1)	216(3)	112(1)	609(5)	168(3)	45(1)	-	36(1)	-	145(2)	140(1)	-	23(1)	253(4)	26(1)	4726(39)	1082(13)	NA

*ppm (one ppm of ARG is defined as one base pair of ARG sequence per million base pairs of nanopore 2D reads; Yang et al., 2013) was used to measure the concentration of resistance genes. The number of valid ARG-hits on nanopore 2D reads corresponding to the concentration was shown in brackets. Carrier species were sorted descendingly based on their prevalence within the community. Abbreviations of antibiotic resistant gene types are: Ami, Aminoglycoside; β-lac, beta-lactamase; Chl, chloramphenicol; Mac, Macrolide-lincosamide-streptogramin; Tet, tetracycline; Bac, Bacitracin; Ble, bleomycin; Fosf, Fosfomycin; Fosm, Fosmidomycin; Kas, Kasugamycin; Poly, Polymyxin; Quin, Quinolone; Rif, Rifamycin; Sulf, Sulfonamide; Trim, Trimethoprim; Mult, multidrug resistant genes*.

“*-” resistance gene is not detected*.

* *resistance genes against the antibiotics used in the isolation of coliform bacteria*.

a *Abun: relative abundance of the carrier species within the 511 Nanopore 2D reads that could be phylogenetic assigned at species level*.

*NA, data not available*.

**Figure 1:**
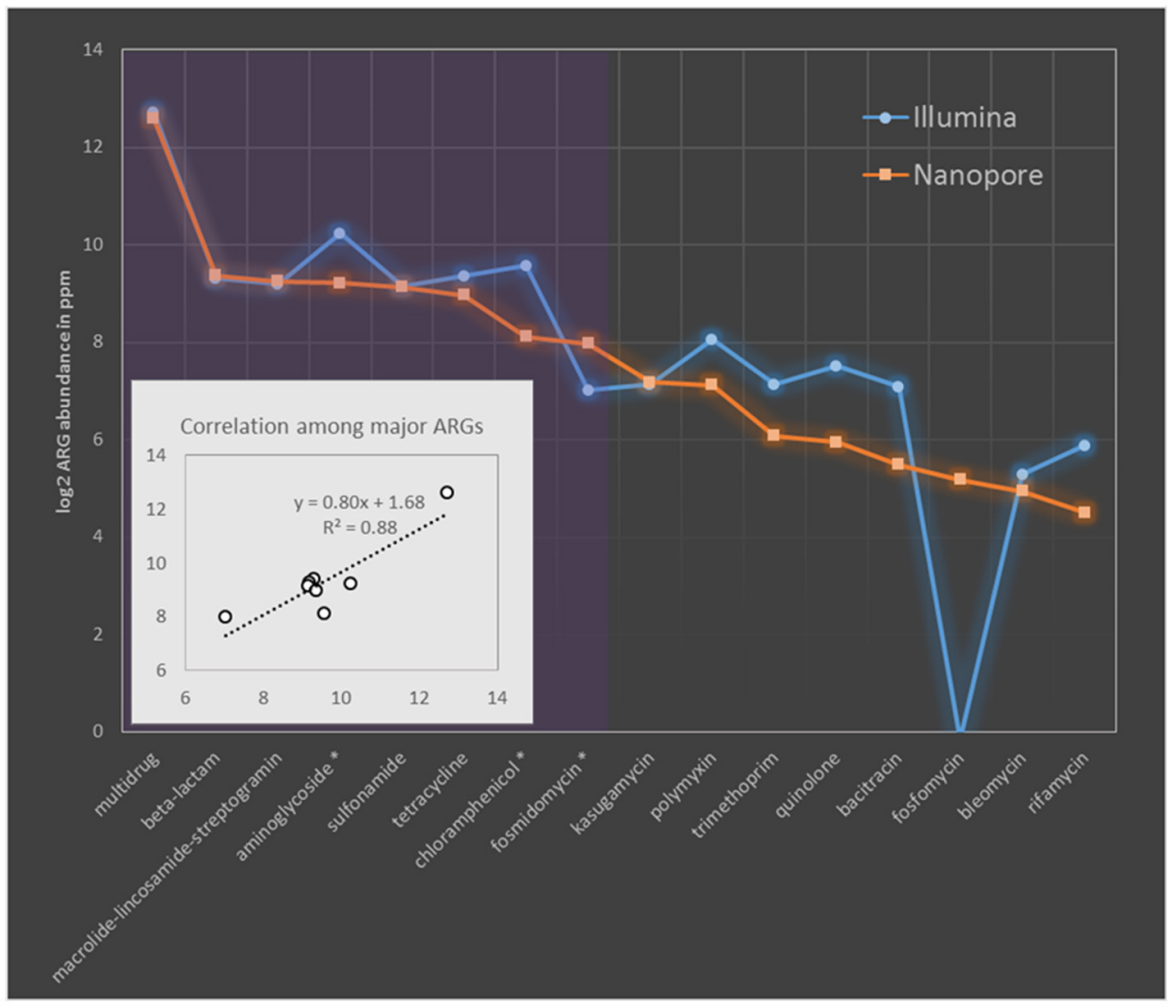
Comparison of MAR coliforms resistome quantification based on Nanopore 2D reads and Illumina short-gun sequencing. The abundance of ARGs was compared in ppm. ARG types are sorted descendingly according to their relative abundance in nanopore dataset. Purple region covers the major ARG types which took more than 2% of the ARG concentration (in term of ppm) in nanopore dataset. Names of major ARG types showing larger than 1.5 times fold change between Nanopore and Illumina quantification was indicated with asterisk in the x axis. Sub-figure in the left bottom corner shows the linear correlation among major ARG types quantified by these two platforms.

The resistome profile revealed by nanopore sequences was then compared to that based on Illumina short reads. To make the results comparable, ARG abundance was compared in term of ppm as previously described, briefly one ppm of ARG is defined as one base pair of ARG sequence per million base pairs of sequence data (Yang et al., 2013). The resistome based on these two sequencing platforms were principally consistent evidenced by the same ARG types covered and comparable quantification with Pearson correlation efficiency of 0.88 (*p*-value of 0.002) among major ARG types (Figure 1). Evident differences (larger than 1.5 times fold change) were observed only in three major ARG types (showing prevalence larger than 2.0% of the total ARGs) of aminoglycoside, chloramphenicol and fosmidomycin. Among them aminoglycoside and chloramphenicol, respectively showed Illumina quantification 1.7 and 2.3 times higher than that based on nanopore, while quantification of fosmidomycin was 2.3 times higher by MinION sequencing (Figure 1). Previous resistome quantification based on Illumina datasets has reported a variation within 15% between biological replicates (Flach et al., 2017). Though the data was not available in literature for degree of resistome variation between MinION runs, a relatively comprehensive and stable ARG collection could be achieved by MinION sequencing (Schmidt et al., 2016). Thereby, we speculated the >1.5 times discrepancy observed here should represent deviation beyond the bounds of experimental error. These discrepancies should be originated from the different ARG prediction algorithms adopted by these two sequencing platforms as MinION methods was based on nucleotide search while Illumina algorithm was based on similarity search of amino acids. Additionally these differences remained when we compared Illumina and nanopore at same data size (Table S8).

Another thing to notice is that ARG genotypes (indicated by ARG subtypes) revealed by Illumina dataset tends to be more diverse than that based on nanopore quantification (Table S9), especially for β-lactam resistance genes, 21 genotypes were identified in Illumina dataset while nanopore only reported 8 (Table S9). Such conservative subtype prediction of nanopore reads was enabled by their long alignment length to ARG reference sequences. For example, if two ARG genotypes with overlapped region, both showed valid alignment to a MinION read, then only the genotype showing the highest bit score to nanopore read will be reported in nanopore quantification, while to the contrary, the multiple Illumina short reads each covering a short fragment of the same genomic region, may hit to different ones of these genotypes based on regional similarity and thus reported both genotypes in Illumina results. These differences in diversity estimation could be especially evident for ARG types, like beta-lactam resistance genes, in which genomic overlaps are common among subtypes. As shown in Table S9, genotypes of beta-lactam reached to as high as 228 when the whole Illumina dataset was used for quantification. To further check if MinION sequencing may be too conservative to give a comprehensive ARG subtype detection, five ARG subtypes which were detected only by either nanopore or Illumina dataset, were selected for PCR verification. As shown in Figure S6, two subtypes exclusively identified by nanopore platform were successfully amplified using the genotype-specific primers designed in this study (Table S4), demonstrating accurate genotyping could be achieved by the applied ARG annotation pipeline of nanopore dataset and Illumina methods failed to detect these ARG subtypes. However, the other three subtypes not detected by nanopore platform also got amplified suggesting the missing of actual genotypes in nanopore dataset. The missing of subtypes by MinION could result from limited sequencing depth (Figure S7), though it is sufficient to represent most resistome of the coliform community.

### Whole genome sequencing of the multidrug-resistant COL1 clone

To resolve resistance development in MAR coliform bacteria by whole genome sequencing using MinION nanopore technology, one of the MAR coliform isolates, the COL1 clone, was randomly picked for deeper MinION sequencing and independent Illumina sequencing. Hybrid assembly of MinION sequences and Illumina short reads resulted in assembly of five contigs with the longest one of 3.3 Mb, almost 70% of the whole genome. By filtering out contigs with inconsistently low coverage (coverage ranged from 1.0 to 6.5) which were generated by excessive overlap assembly of MinION reads (Loman et al., 2015), the final COL1 assembly contained a 4.9 Mb chromosome with average GC content of 51.0% and average coverage of 297.0 ± 16.9.

The COL1 chromosome was predicted to harbor seven rRNA operons consist of consecutive single copy of 5S, 23S, and 16S rRNA genes (Figure S8). 4,686 coding sequences (CDS) were predicted on the COL1 genome with coding density of 86.9%; approximately 23.1% (1,083/4,686) of the CDS of COL1 strain possessed no clear biological function. Meanwhile, the five identical copies of 16S rRNA gene in the COL1 strain showed the closest affiliation (99% similarity over 100% length) to *Shigella flexneri* 2a str. 301, the causative agent of shigellosis (Clark and Maurelli, 2007). What is more, the coding sequences of the COL1 strain showed the highest average nucleotide identity (ANI) of 98.7% to that of the human pathongen strain *Shigella boydii* Sb227 (Yang, 2005).

**Figure 2:**
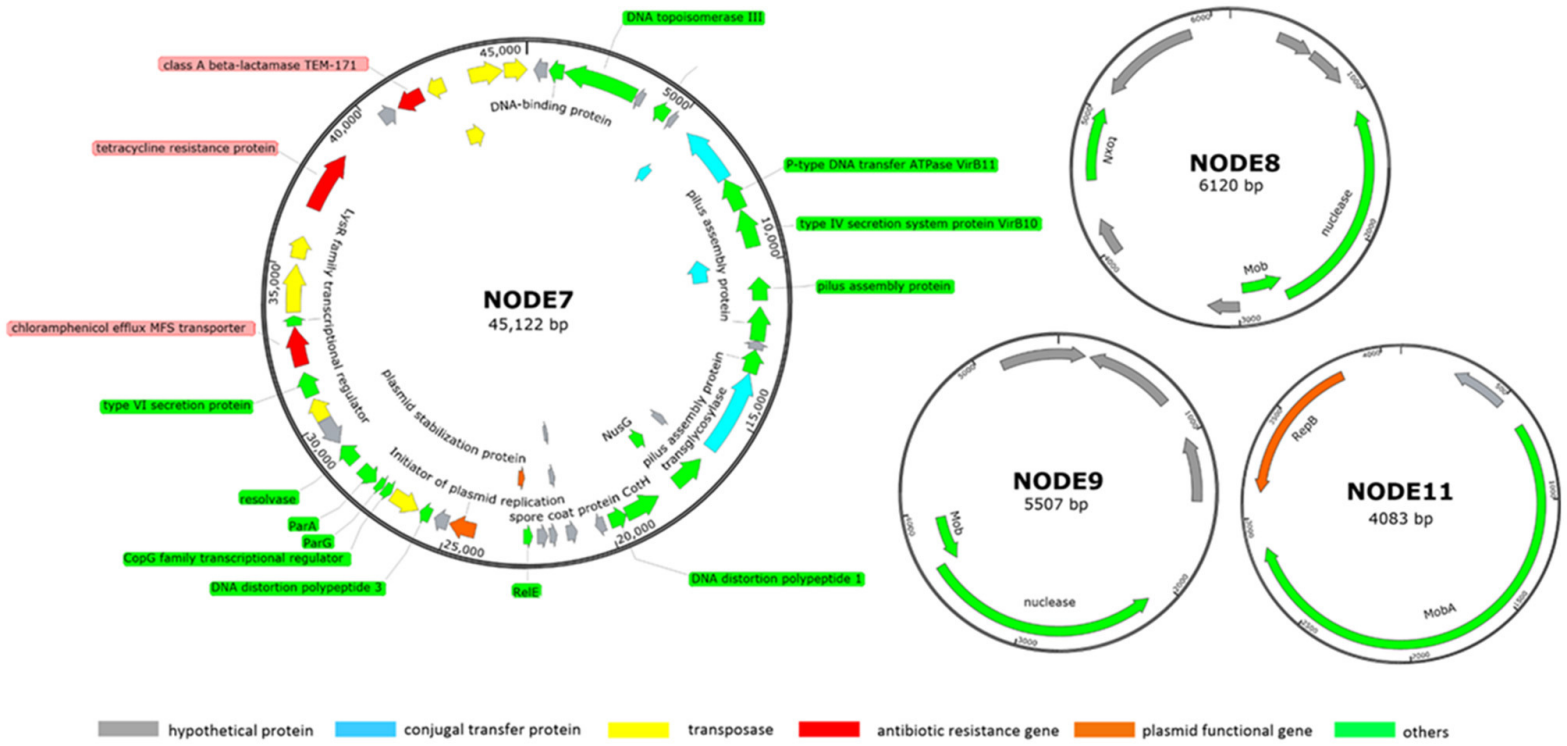
Subset of manually curated plasmids assembly (NODE7, NODE8, NODE9, and NODE11) from strain COL1. The large plasmid NODE5 is not displayed because it is virtually identical to that reported by Liu et al. (2015, p. 033). Circles displayed (from the outside) (1) coordinates in kilobase pairs. (2) protein-encoding ORFs predicted and annotated based on the NCBI categorization. Genes displayed are colored by category as follows: red, ARGs; orange, plasmid functional genes; blue, genes encoding conjugal transfer protein; yellow, transposase genes; gray, genes encoding hypothetical proteins; green, other functional genes.

Five more contigs were identified as plasmid sequences in the hybrid assembly including: two large plasmids of NODE5 (160 kb) and NODE7 (40 kb) whose circular structure was confirmed by PCR spanning; three scaffolds of NODE8 (6 kb), NODE9 (5 kb) and NODE11 (4 kb) showing circular structure with overlapped bases at start and end respectively of 77 bp, 77 bp and 78 bp (Figure 2). Estimated based on plasmid coverage values (650× for NODE5, 416× for NODE7, 509× for NODE8, 466× for NODE9, and 656× for NODE11) against average chromosome coverage (279×), the COL1 strain carries 2.3 copies of NODE5, 1.5 copies of NODE7, 1.8 copies of NODE8, 1.7 copies of NODE9, and 2.4 copies of NODE11.

### Source of antibiotic resistance in COL1 strain

Noteworthy, this MAR COL1 strain shares plasmids with strains showing potential health threats to pigs and human. The plasmid of NODE5 (160 Kb, containing resistance against tetracycline) and NODE11 (41 Kb,) were respectively identical to p3PCN033 (99% similarity over 98% of the length) and p2PCN033 (99% similarity over 100% of length) of porcine extraintestinal pathogenic *E. coli* PCN033 (Liu et al., 2015, p. 033). Noteworthy, NODE11 also showed high identity to plasmids of several pathogenic *E Coli* strains including: isolate from infectious bloodstream *E. Coli* SF-468 (plasmid pSF-468-4, 99% similarity over 98% of length) (Stephens et al., 2015) and putative urine virotype *E. Coli* E35BA (plasmid pE35BA, 99% similarity over 100% of length) (Lanza et al., 2014).

Next, the source of resistance was tested for the COL1 strain. First, plasmids of the COL1 strain were extracted and transformed to antibiotic sensitive component *E. coli* DH5α cells and grew overnight in LB medium modified by various antibiotics (Table S1). Resistant cells only grew on LB plates containing 100 μg/mL of ampicillin. Among them, cells carried only exotic plasmid NODE7 (40 kb) was found, confirming ampicillin resistance was introduced by this plasmid. Therefore, plasmid NODE7 could act as the source for ampicillin resistance of COL1 strain. The 160 kb plasmid NDOE5 should serve as one of the source for tetracycline resistance in COL1 strain because plasmid (of *E. coli*. PCN033) virtually identical to it (99% similarity over 98% of length) had been tested to resistant to tetracycline (Liu et al., 2015).

Besides resistance on plasmids, chromosomal resistance genes were also investigated. A complete set of ARGs against the antibiotics used for isolation could be identified in the genome of COL1 strain (Table 2). Macrolide phosphotransferase enabling erythromycin resistance was encoded only on the chromosome but not on plasmids (Table 2). Given the high concentration of antibiotics used for isolation of COL1, it was quite evident that plasmids may contribute to resistance of COL1. However, chromosomal resistance mechanism could also contribute part of the overall resistance phenotype. Therefore, the analysis based on sequencing may not sufficient to differentiate the plasmid and chromosomal sources of resistance.

**Table 2:** Copy of antibiotic resistant gene identified on the chromosome and plasmids of COL1 strain.

**ARG type**	**Chromosome**	**NODE5**	**NODE7**
Beta-lactamase^a^	2	1	1^*^
Chloramphenicol^a^	1	1	1
Macrolide^a^	1	-	-
Aminoglycoside^a^	1	3	-
Tetracycline^a^	1	1	1
Acriflavine	6	-	-
Bacitracin	2	-	-
Fosmidomycin	1	-	-
Fusidic_acid	1	-	-
Multidrug	4	-	-
Polymyxin	1	-	-
Sulfonamide	-	1	1
Trimethoprim	-	1	-
Others	8	1	-

*Only plasmids with at least one ARGs identified were includes*.

a *Antibiotic resistant genes against antibiotics applied for coliform isolation. Beta-lactamase against ampicillin; chloramphenicol against chloramphenicol; macrolide against erythromycin; aminoglycoside against kanamycin and tetracycline against tetracycline*.

* *Resistance have been validated by experiment*.

“*-” not detected*.

**Figure 3:**
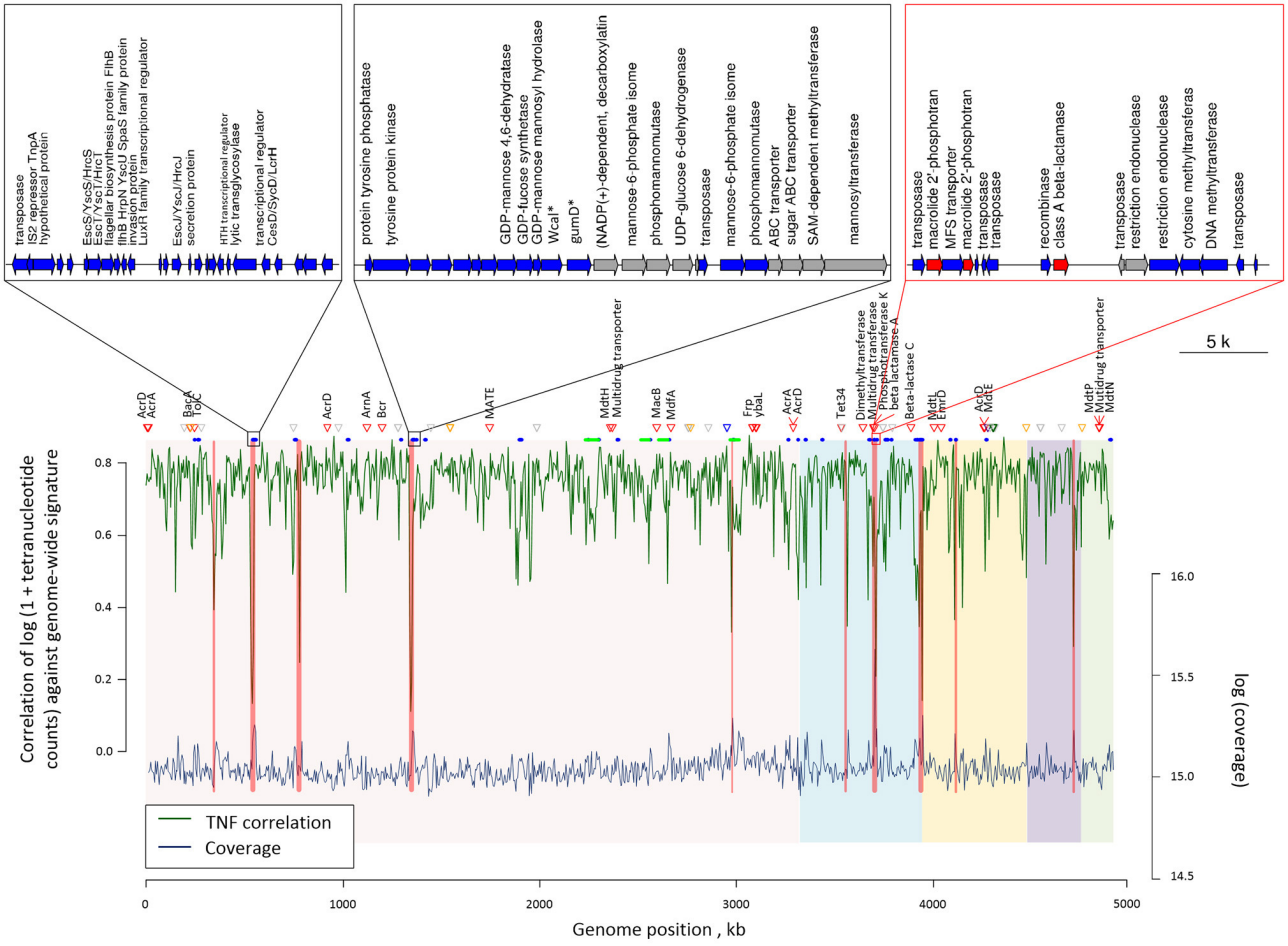
Genome plot showing the tetranucleotide frequency (green line) and coverage (blue line) across the COL1 chromosome. Genomic regions with showed significantly verified (*p* < 0.05) tetranucleotide frequency correlation and coverage are highlighted in red bands. Red triangles show the position of antibiotic resistant genes, while orange, blue, green and purple triangles respectively represent metal resistance genes against Zinc, Copper, Nickel, and Arsenic. Gray triangles show other metal resistant genes. Blue and green segments below respectively represent the position of gene islands and phage operons observed. The five chromosomal contigs of COL1 assembly were colored differently in background. Gene arrangement was shown for the three gene islands with the most inconsistent tetranucleotide frequency correlation and coverage. Genes are colored according to their categories: blue, island related genes; red, ARGs; gray, other genes. Gene abbreviations used in the plot: Wcal: colanic acid biosynthesis glycosytransferase; gumD: undecaprenyl-phosphate glucose phosphotransferase.

Sixty-one genomic islands with average length of 11,650 bp were identified across the COL1 chromosome (Dhillon et al., 2015). Tetranucleotide frequency (TNF) of genomic DNA sequences of an isolate is usually highly conserved, and differences in the TNF are mainly due to the intercellular genetic exchange and would be clue for the horizontal gene transfer (Noble et al., 1998). As shown in Figure 3, there are seven genomic islands on the COL1 genome showed significantly divergent (z-score beyond critical z-score at 95% confidence interval, *p* < 0.05) TNF signature and coverage than the rest of the chromosome. Noteworthy, one of these horizontally acquired islands encoded antibiotic resistance operon consist of ARGs against erythromycin (macrolide 2′-phosphotransferase) and ampicillin (class A broad-spectrum beta-lactamase). A complete set of transposase and recombinase was identified near these resistance genes confirming the exogenous origin of these ARGs. More interestingly, the same class A beta-lactamase (accession: AAZ79402.1) was shared among COL1 chromosome and its plasmids (NODE5 and NODE7). The co-occurrence of this resistance gene on the chromosome and plasmids of COL1 strain, especially the plasmid shared with porcine bacterial pathogen (NODE5 shared with porcine pathogen *E. Coli* PCN033), indicated a potential chromosomal integration of exogenous resistance gene obtained through plasmid exchange between antibiotic-resistant bacteria across geographically distant niches (COL1 strain was isolated from WWTP of Hong Kong, while the porcine pathogen PCN033 was isolated from brain of pig grown in Human province, China). Moreover, a class A beta-lactamase (*TEM171*) gene located on both chromosome and plasmids of COL1 was also identified on the genomic fragment of the dominant population of *Klebsiella pneumoniae* (Table 1), an important pathogen in nosocomial infections (Podschun and Ullmann, 1998). The finding of inter-phylogeny shared class A beta-lactamase (*TEM171*) here, plus a previous reported identification of the same resistance gene among functional selected libraries across different ecological gradients (Pehrsson et al., 2016), indicated the wide distribution of this resistance machinery across environmental habitats.

Additionally, four complete phage structures of Phage_Entero_mEp460, Phage_Escher_pro147, Phage_Entero_Fels2, and Phage_Entero_lambda were identified on COL1 chromosome, but none of them carried resistance gene (Table S10), suggesting the resistance of COL1 strain might not be gained via resistance transduction by bacterial phage infection. 41 metal resistance genes enabling cellular tolerance to a variety of heavy metals including nickel, zinc, copper, arsenic, silver, and mercury, were identified on the COL1 genome (Figure 3). Such co-existence of metal resistance may favor the indirect selection of antibiotic resistance of COL1 under metal pressure (Baker-Austin et al., 2006; Seiler and Berendonk, 2012). However, since no metal resistance gene was identified in the same operon of ARGs on the chromosome (Table S11), resistance toward antibiotic and heavy metal should be regulated independently in COL1 strain.

## Discussion

In the present experiments, genotype resistance prediction based on Nanopore technology was found to be reliable as long match to reference sequences could be achieved and the quantification based on MinION sequencing was largely consistent to that based on Illumina sequencing platform. However, difficulties remain in assigning phylogeny for ARG-containing nanopore reads as marker genes may not be located in close genomic proximity of ARGs. Furthermore, although examination of water sanitation currently undergoes protocols that tend to reduce diversity, such as coliform bacteria test, future development of real-time monitoring will surely involve reduction in culture time, and potentially increase the levels of diversity detected in the sample. Consequently, improvement on sequencing throughput is critical to develop a framework extensible to many species for MinION sequencing. By the publication of this work, the updated chemistry R9.4 has been reported to be able to produce 2D dataset larger than 200 Mb in a single 24 h MinION run (Cornelis et al., 2017). Furthermore, improved accuracy of 1D reads (around 90% in R9 chemistry) coupled with the rapid 1D sequencing protocol has shown complete species level diversity recovery of mock communities based on muli-locus ribosomal RNA genes amplicon sequencing (Benitez-Paez and Sanz, 2017). These rapid improvements in both throughput and accuracy of MinION sequencing demonstrated its potential as a powerful tool for environmental resistome quantification. Thereby, the appraisal of limited sequencing accuracy and output of MinION sequencing applied here should not in any way diminish its value as pioneer framework to identify the most appropriate usage of this technology in antibiotic resistance detection and host tracking for water samples.

The resistance detected here was mostly focused on the resistance genotype. We did not verify the function of each ARGs identified in the target coliforms. Although the sequenced based antibiotic resistance prediction was widely used in different systems, we must recognize that the existence of ARGs does not represent their contribution to the resistance phenotype for their hosts. For example, detection of macrolide phosphotransferase gene here would not indicate its resistance phenotype contribution. Additionally, for coliform bacteria, because of its gram-negative nature, the outer membrane barrier and efflux pumps make the macrolides (relative large molecules and hydrophobic) much less active against Gram-negatives (Vaara, 1993). Therefore, the expression information of related resistance genes is needed to further determine the resistance phenotype of these identified ARGs.

Additionally, one thing we must notice is that ARGs summarized in the category of multidrug resistant and others would not contribute to the resistance phenotype directly. For example, gene detection of multidrug transporter does not convert to resistance phenotypes. In each gram-negative bacterium, despite the presence of multidrug transporter homologs, only a few of them contribute to resistance phenotypes (Higgins, 2007). The *omp*F and *omp*36 genes are expected in wild type susceptible *E. coli* or *K. pneumonia*. Absence or reduced expression of these genes is associated with resistance, while their presence does not contribute to the resistance phenotype directly (Thiolas et al., 2004). Similarly, the presence of *hns* genes, which is classified in the category of “others” listed in the SARG database, does not explain resistance while its expression status would contribute the resistance phenotype for their host (Li et al., 2016).

To further explore the resistance development in MAR coliform bacteria, the COL1 clone was picked out for deeper MinION sequencing and independent Illumina sequencing. The taxonomic analysis results suggested this opportunistic pathogen COL1 strain shall process genotypic features that may impose potential risk to human health though its pathogenicity and virus factor cannot be tested here. Additionally, the multiple instances of plasmid sharing between porcine/human pathogens and this environmental coliform strain from wastewater treatment processes demonstrated a potential exchange of genetic material including ARGs between environmental bacteria and pathogens, which reinforced the speculation that microbiota in domestic wastewater treatment systems may serve as an important reservoir of resistance genes available for exchange with clinical pathogens.

Though reliable resistance gene prediction could be carried out based on the long and erroneous Nanopore reads, the limited sequencing throughput was the main constraint of MinION sequencing for resistome prediction of environmental samples. However, the rapid optimization and development of the MinION platforms may solve such problem in the near future. With the described approach, the resistome profiles could be clarified much faster compare to using the traditionally high-throughput sequencing platforms.

## Author contributions

YX and AL carried out the experiment, data process, as well as manuscript drafting. YD contributed in gene islands annotation. XJ prepared the nucleotide sequences of the SARG database and carried out the phage identification of COL1 strain. LL contributed to the metal resistant gene identification of COL1 strain. TZ coordinated experiment design and manuscript writing.

### Conflict of interest statement

The authors declare that the research was conducted in the absence of any commercial or financial relationships that could be construed as a potential conflict of interest.
