# Supplementary material for: MinION Nanopore Sequencing Enables Correlation between Resistome Phenotype and Genotype of Coliform Bacteria in Municipal Sewage
Source: Front Microbiol. 2017 Oct 31;8:2105. doi: 10.3389/fmicb.2017.02105 (PMC5671560; doi:10.3389/fmicb.2017.02105)

Supporting Information

MinION nanopore sequencing enables rapid resistome profiling and antibiotic resistance island reconstruction of coliform bacteria in municipal sewage

Yu Xia †, An-Dong Li †, Yu Deng, Xiao-Tao Jiang, Li-Guan Li, Tong Zhang *

Methods

Library preparation for MinION sequencing

Briefly, 1.5μg extracted DNA was diluted into 81 μl nuclease-free water and sheared using a Covaris g-TUBE by centrifuging the DNA solution through the column in both directions at 6000 rpm for 1 min (Eppendorf 5424) (Sample was centrifuged for another 1 min if the DNA solution had not passed through completely). The shared DNA fragments were end-repaired using the End Repair Moudle (NEB, cat. No. E6051A) by adding 5μl enzyme mix and 10 μl reaction buffer into the sheared DNA solution and then incubating at room temperature for 20 min. The end repaired DNA fragments were then purified by adding 100 μl resuspended AMPure XP beads (Beckman Coulter Inc., cat. no. A63880) at room temperature and mixing by hand rotation for 5 minutes. The DNA binding beads were pelleted on magnet (Invitrogen MagnaRack, cat. no. CS15000) and washed twice with 200μl fresh 70% ethanol without disturbing the pellet. DNA was eluted in 26 μl of nuclease-free water after the washing. Next, the dA-tailing was then performed using NEBNext dA-Talling Module (New England BioLabs, cat. no. E6054A) in a total volume of 31μl by adding 3μl buffer and 2μl enzyme to the clean DNA. The system was then mixed by inversion and incubated at 37 ℃ for 10 min. The library was purified for another time using the AMPure XP beads and eluted with 31 μl nuclease-free water. A ligation reaction was then performed by adding 50 μl Blunt/TA DNA ligase (New England BioLabs M0367L), 10 μl adaptor mix, 10 μl HP adaptor into the dA-tailed DNA. The reaction was gently pipetted up and down to mix and incubated at room temperature for 10 min. The adapted-ligated DNA was purified using the prepared MyOne C1 beads (Invitrogen Life Technologies) and finally eluted with 25μl elution buffer.

MinION sequencing

During the last half-hour of library preparation, MinION device was connected to the computer via USB3. After inserting flow cell (MAP005 Chemistry) into the MinION, a flow cell quality control protocol was run to assess pore activity: 180 single pores were detected for the flow cell used. Next, flow cell was equilibrated by pipetting two aliquots of 500 µl priming buffer (prepared by: 500 µl running buffer, 475 µl nuclease-free water and 27 µl fuel mix) into the flow cell, incubating for 10 min after each addition. Immediately after the library preparation, 6 µl of the prepared library was mixed with 75 µl running buffer, 65 µl nuclease-free water and 3 µl fuel mix in a fresh tube. The mixed sequencing library was gentle loaded into the flow cell via sample port using a vertical 1000 µl pipette and tip in a continuous flow avoiding introducing air bubbles or disturbing the sensor array. The 24 h sequencing protocol was selected for sequencing.

PCR system and condition used for close the circular structure of NODE5 and NODE7

The 50 µl PCR system included: 25 µl of Takara reaction mix, 1 µl extracted plasmid, 1.5 µl (10 µM each) primer mix and 21µl of nuclease free water. PCR condition were: initial denaturation at 95 ℃ for 2 min, followed by 35 cycles of (denaturation at 95 ℃ for 30 s, annealing at 55 ℃ for 30 s, and extension at 72 ℃ for 4 min), and then final extension at 72 ℃ for 10 min. The circular structure of NODE5 and NODE7 could be confirmed as PCR products of 7kb and 5kb were respectively amplified by the designed primers.

Quality-control of Illumina reads

Short paired-end reads delivered by Illumina sequencing (Q20 ≥ 85% and Q30 ≥ 80%) were trimmed with the following parameters: (1) remove reads with adapter, (2) remove reads containing N in more than 5% of the bases, (3) remove reads with Q ≤ 19 base percentage larger than 15%. 2.6% of raw reads were filtered during the quality control step resulting in 28,963,774 clean reads.

Tables and Figures

Table S1. List of antibiotics used for multidrug-resistance bacteria isolation

Antibiotics name	Abbreviation	Class	Cellular target	Dosage, mg/l
Ampicillin	Amp	Beta-lactams	Cell wall	100
Tetracycline	Tet	Tetracycline	Protein synthesis, 30S	10
Kanamycin	Kan	Aminoglycoside	Protein synthesis, 30S	50
Erythromycin	Ery	Macrolide	Protein synthesis, 50S	100
Chloramphenicol	Chl	Chloramphenicol	Protein synthesis, 50S	16

Table S2. DNA concentration after each step of MinION library preparation.

Sample name	Preparation step	Volume µl	DNA concentration ng/µl	DNA weight µg
COL1 strain	Start preparation	100	15.0	1.5
	DNA shearing and end-repair	25	56.7	1.4
	dA-tailing	30	37.2	1.1
	Adaptor-ligation	25	6.0	0.15
Coliform bacteria	Start preparation	100	12.0	1.2
	DNA shearing and end-repair	25	53.6	1.3
	dA-tailing	30	32.0	0.96
	Adaptor-ligation	25	5.8	0.14

Table S3. Primers designed to complete the circular structure of NODE5 and NODE7

Target plasmid	Forward primer	Reverse primer
NODE5	CTGGCTGCTACACCATGTTGC	CTACCGGGCTAATGAAACACG G
NODE7	TCTTCTTGTATCGCACTGTCTTGTTAC	GGTGACCCCCCCACAGAACAAGAG

Table S4. Primers designed to validate the ARG subtypes. Primer 3 and 4 are targeting ARG subtypes only identified by NanoPore dataset, while the other three are for that exclusively detected by Illumina platform.

No.	Gene ID	Forward Primer	Reverse Primer	Target length, bp	Targeted ARG subtype
1	AAW82909	CCCGCTGGGTGCAAA	TTAGCCAGCATCACGATAC	331	Class C beta-lactamase
2	ABS58643	GCGCACTGGCTGTGA	TCACTGCCGTTAACGATGA	388	Class C beta-lactamase
3	DQ286729	CAGCAATGGCAACAA	TAATCAGTGAGGCAC	306	beta-lactam_TEM-55
4	KF240809	CAAACGGCGAACGTA	ATCAGCTTATTCATCGC	341	beta-lactam_CTX-M-142
5	YP002806354	ACAGCAAACGTTGTTT	TTTGTCGTTGATATCAC	574	class C beta-lactamase

Table S5. Statistics of Nanopore and Illumina datasets of coliform bacteria and COL1 strain.

Sample	datasets	Reads number	Median length (bp)	Max length (bp)	Total length (Mbp)	Median Accuracy (Phred score)	Median Accuracy based on mapping to Illumina assembly	Estimated Coverage
Coliform isolates	Nanopore 2D reads	3,535	2,503	12,445	11.1	90.5% (10.2)	NA	NA
	Illumina Hiseq2500	26,543,006	125	125	3,164.2	99.9% (30)	NA	NA
COL1 strain	Nanopore 2D reads	8,512	5,796	18,582	46.9	91.0% (10.5)	84.6%	10×
	Illumina Hiseq2500	28,963,774	125	125	3,452.8	99.9% (30)	NA	720×

NA: estimation is not available

Table S6. Number of nanopore 2D reads could be classified at various taxonomic levels. The corresponding classification ratio was shown in brackets.

	**Number of all nanopore 2D reads**	**Number of ARG-carrying nanopore 2D reads**
total	3535	97 (2.7%) 1)
classified at phylum level	852 (24.1%)	48 (49.5%*)
classified at class level	852 (24.1%)	48 (49.5%*)
classified at order level	852 (24.1%)	48 (49.5%*)
classified at family level	850 (24.0%)	48 (49.5%*)
classified at genus level	830 (23.5%)	47 (48.5%*)
classified at species level	511 (14.5%)	31 (32.0%*)

1): 2.7% was the ratio of ARG-carrying nanopore reads in percentage of total 3535 nanopore 2D reads.
*: classification ratio of ARG-carrying nanopore 2D reads was calculated in percentage of the 97 ARG-carrying nanopore 2D reads

Table S7. Phylogenetic composition of the coliform culture based on marker gene searching using Nanopore 2D reads.

Genera 1)	Number of Nanopore reads	Percentage 2)	Genera	Number of Nanopore reads	Percentage*
Klebsiella	241	29%	*Pantoea*	10	1%
Enterobacter	82	10%	*Raoultella*	9	1%
Cronobacter	64	8%	*Xenorhabdus*	7	1%
Escherichia	53	6%	*Aggregatibacter*	6	1%
Yersinia	46	6%	*Providencia*	6	1%
Citrobacter	45	5%	*Punalikevirus*	5	1%
Candidatus Moranella	29	4%	*Siphoviridae noname*	5	1%
Salmonella	28	3%	*Aeromonas*	4	1%
Lambdalikevirus	25	3%	*Enterovibrio*	4	1%
Serratia	24	3%	*Marinobacterium*	4	1%
Dickeya	18	2%	*P22likevirus*	4	1%
Viruses noname	13	2%	*Proteus*	4	1%
Erwinia	10	1%	*Pseudomonas*	4	1%
Myoviridae noname	10	1%	*Rheinheimera*	4	1%

1) Only genera taking more than 1% of the coliform community are shown
2) Percentage within the 852 Nanopore reads that could be phylogenetic assigned at genus level.

Table S8. Comparison of MAR coliforms resistome quantification based on Nanopore and Illumina platforms. Illumina subset dataset is in the same community coverage to that of NanoPore dataset.

ARG types	NanoPore	Illumina subset	Illumina whole dataset
Aminoglycoside	594	1043	1193
Bacitracin	45	86	134
Beta-lactam	664	423	630
Bleomycin	31	86	39
Chloramphenicol	276	967	752
Fosfomycin	36	-	1
Fosmidomycin	251	172	129
Kasugamycin	145	69	138
Macrolide-lincosamide-streptogramin	609	751	578
Polymyxin	140	294	263
Quinolone	62	86	181
Rifamycin	23	37	58
Sulfonamide	558	648	563
Tetracycline	498	674	651
Trimethoprim	68	109	140
Multidrug	6141	6525	6691
Unclassified	1220	1808	1664

-: not detected

Table S9. Comparison of the number of ARG subtypes covered by NanoPore and Illumina platforms. Illumina subset dataset is in the same community coverage to that of NanoPore dataset.

ARG types	NanoPore	Illumina subset	Illumina whole dataset
Aminoglycoside	3	9	13
Bacitracin	1	1	1
Beta-lactam	8	21	228
Bleomycin	1	2	2
Chloramphenicol	2	7	7
Fosfomycin	1	-	1
Fosmidomycin	2	2	2
Kasugamycin	1	1	1
Macrolide-lincosamide-streptogramin	2	4	6
Polymyxin	1	1	1
Quinolone	1	1	2
Rifamycin	1	1	1
Sulfonamide	2	3	3
Tetracycline	3	6	9
Trimethoprim	1	2	10
Multidrug	20	33	45
Unclassified	6	8	8

-: not detected

Table S10. Annotation of genes carried by the four complete phage operons identified on the chromosome of COL1 strain.

Table S11. Annotation of ARGs and metal resistant genes identified on the chromosome of COL1 strain.

Figure S1. Schematic of the study workflow

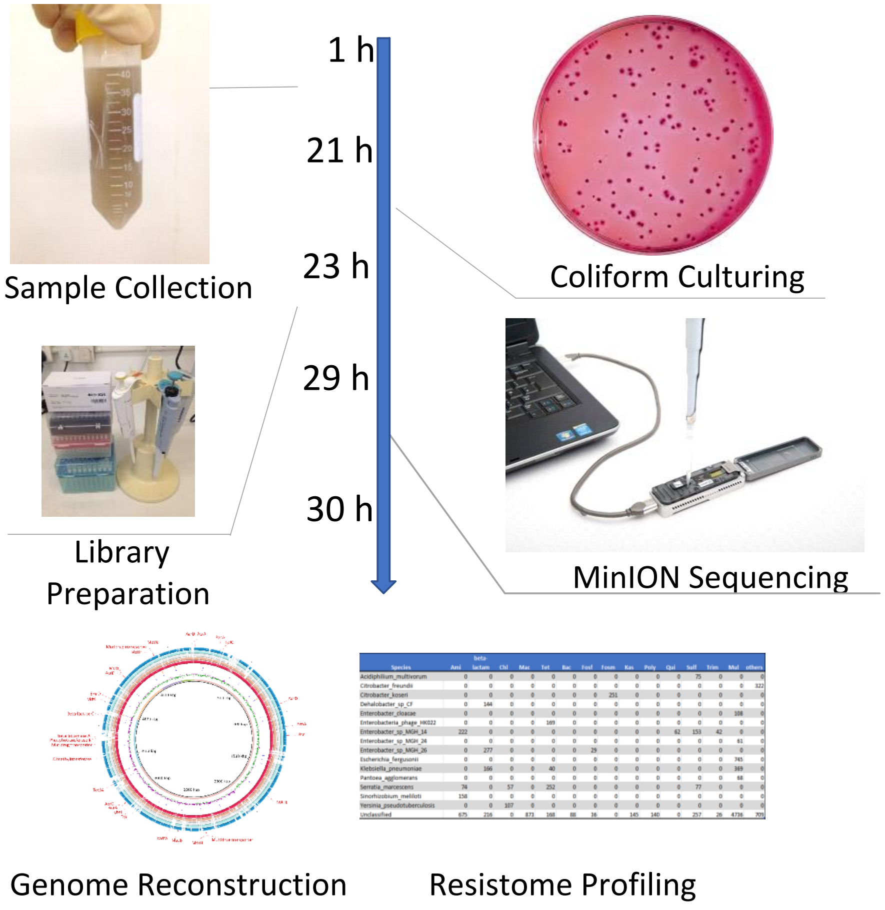

Figure S2. Gel figure for closing circular structure of plasmid NODE5. M1: Lambda DNA/Hind III DNA Marker, M2: DL2000 DNA Marker, N5: PCR product of circular structure closing of plasmid NODE5.

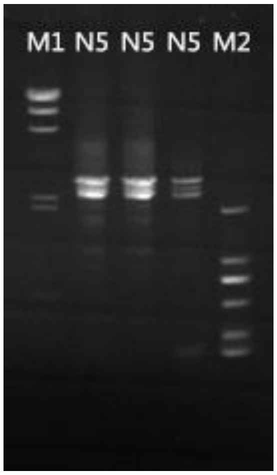

Figure S3. Box-and-whiskers plot of the z-score for inserted (left) and deleted (right) k-mers of 3-6 bp in length, grouped by the proportion of GC content. A higher value of z-score indicates higher-than-expected tendency for insertion or deletion.

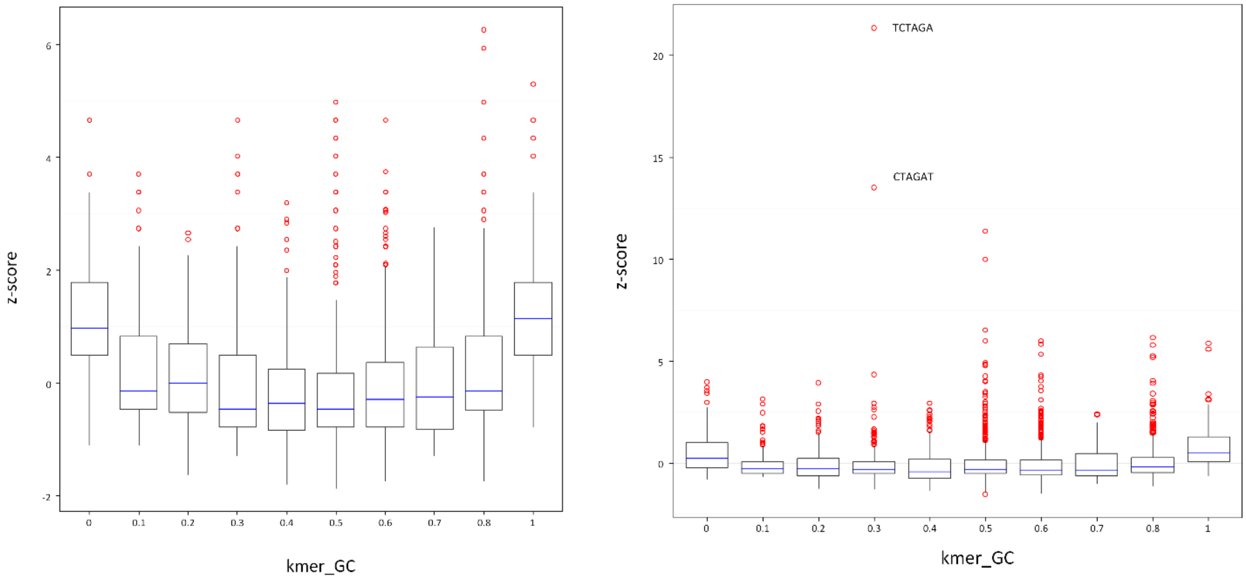

Figure S4 Distribution of Alignment length (shown as bar) and corresponding similarity (shown as triangles) in BlastP results of nanopore 2D reads against reference sequences in SARG database.

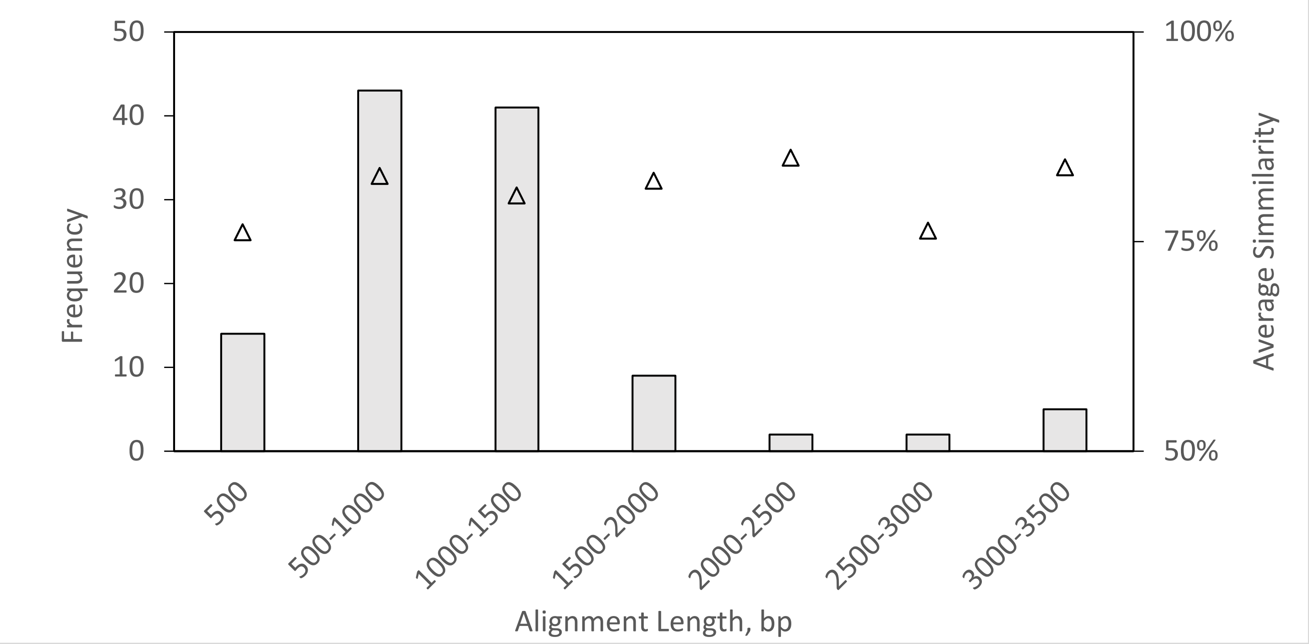

Figure S5 Distribution of various ARG types within the SARG database.

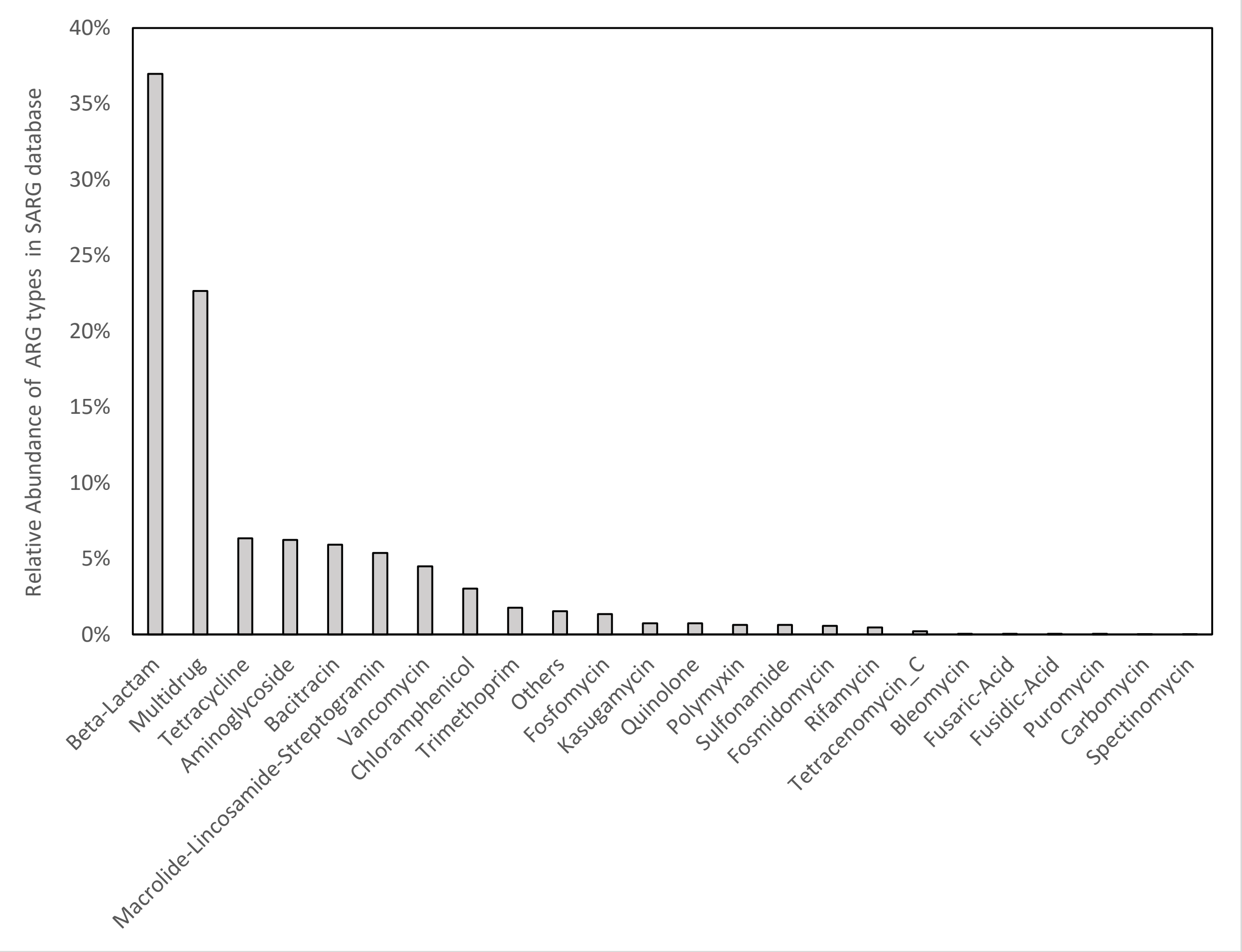

Figure S6 Gel figure for ARG subtypes validation. Band 3 and 4 represent the amplification for ARG subtypes exclusively identified by NanoPore dataset, while the other three bands represent that only identified by Illumina sequencing. Band M stands for marker gene.

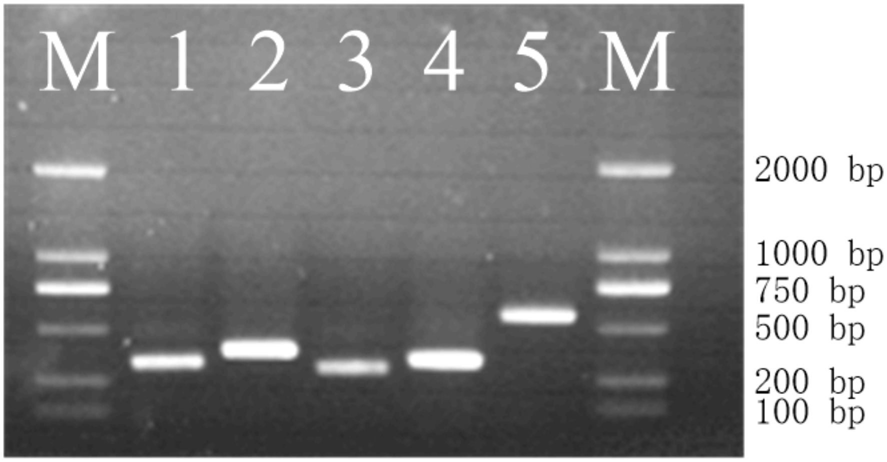

Figure S7 Rarefaction curve of detected ARGs types and subtypes based on nanopore (top figure) and Illumina (bottom) datasets. The blue line represents the number of ARG types detected, while the number of ARG subtypes detected are shown as orange line. The dash line represents the logarithmic regression of rarefaction analysis results and R square (regression coefficient) is shown on the figure beside the regression line.

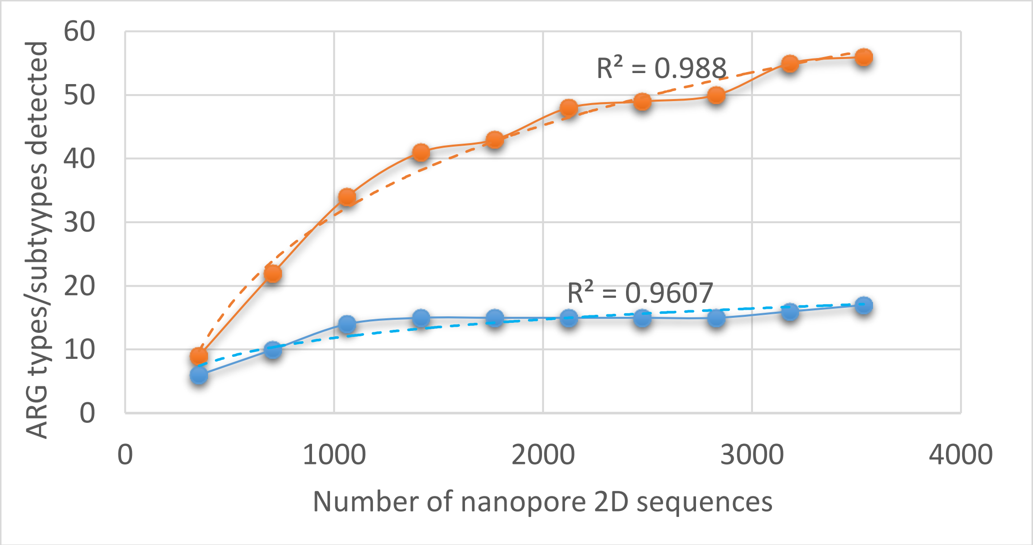

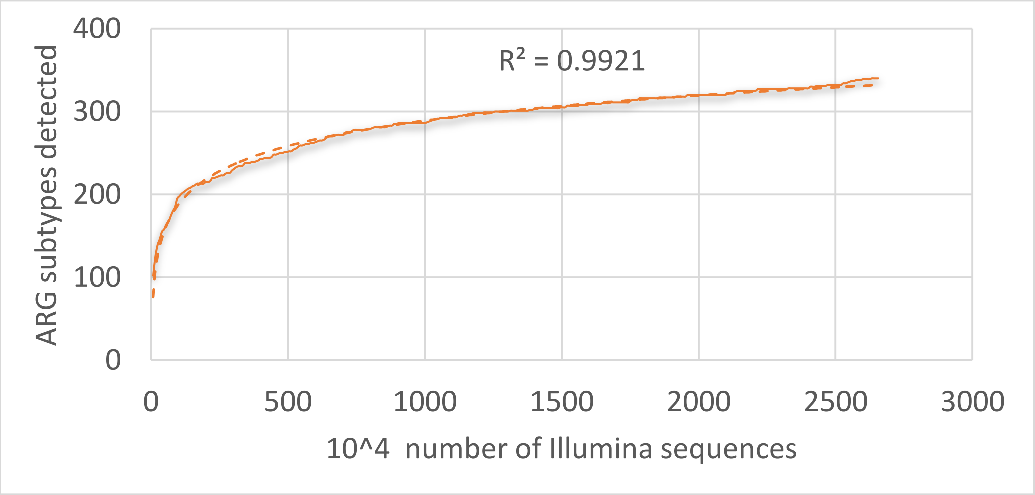

Figure S8 BRIG mapping of COL1 genome to reference genomes. Reference genomes were selected if they showed closest match to 16S rRNA genes (Shigella flexneri 2a, Shigella boydii sb227) or plasmid sequences of COL1 strain (E. coli PCN033, E coli SF468 and E fergusonii ATCC35469). The inner circle represents the five chromosomal contigs of COL1 genome with the same color scheme to that of Figure 1. Genomic positions of ARGs were highlighted in the out most circle.

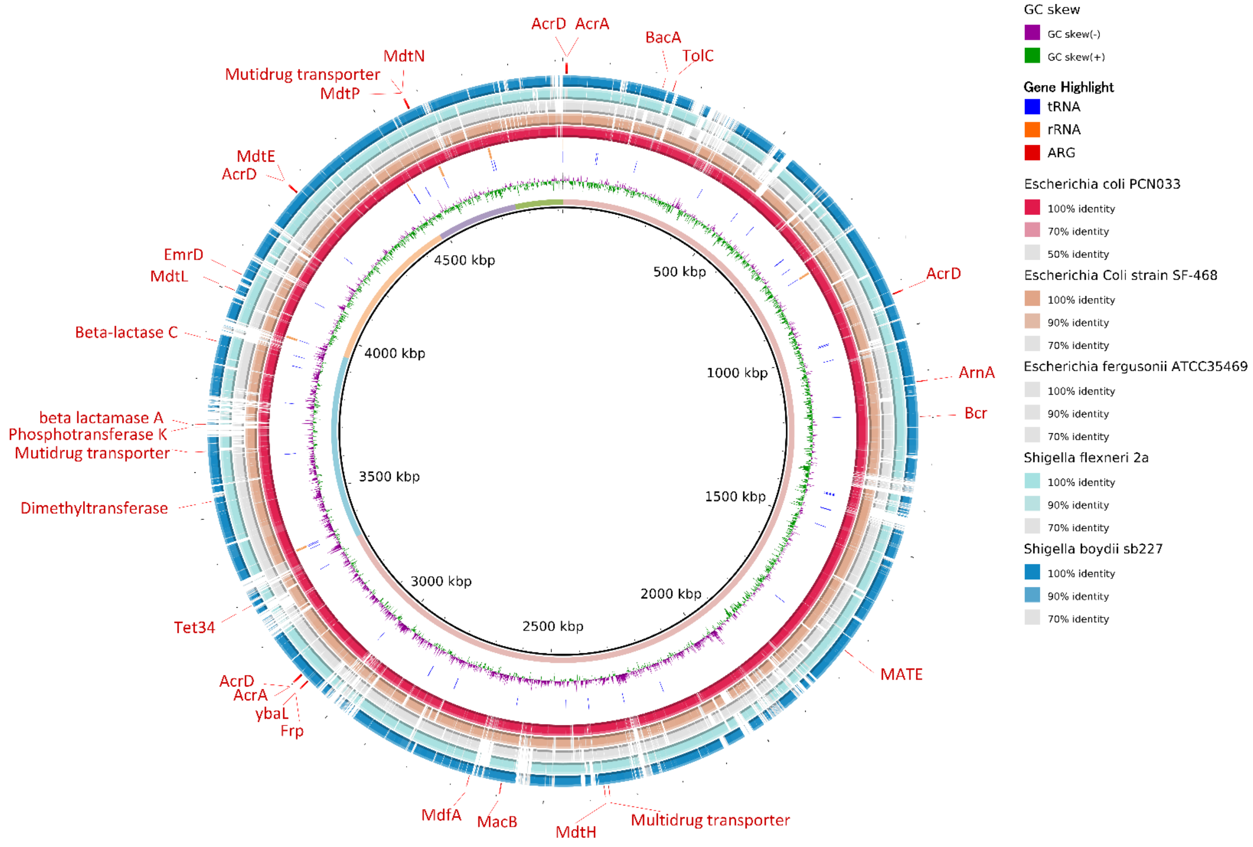

Supplement: Supplementary file 3 [file Presentation1.pdf]
